# Overcoming Obstacles in the Development of Antigen-Specific Immunotherapies for Type 1 Diabetes

**DOI:** 10.3389/fimmu.2021.730414

**Published:** 2021-08-05

**Authors:** Ranjeny Thomas, José M. Carballido, Johnna D. Wesley, Simi T. Ahmed

**Affiliations:** ^1^University of Queensland Diamantina Institute, Princess Alexandra Hospital, Woolloongabba, QLD, Australia; ^2^Translational Medicine/Preclinical Safety, Novartis Institutes for Biomedical Research, Basel, Switzerland; ^3^Type 1 Diabetes, Immunology, & Kidney Disease Research, Novo Nordisk Research Center Seattle, Inc., Seattle, WA, United States; ^4^Strategic Partnerships, The New York Stem Cell Foundation Research Institute, New York, NY, United States

**Keywords:** T1D, autoimmunity, immunotherapy, tolerance, precision medicine

## Abstract

Antigen-specific immunotherapy (ASI) holds great promise for type 1 diabetes (T1D). Preclinical success for this approach has been demonstrated *in vivo*, however, clinical translation is still pending. Reasons explaining the slow progress to approve ASI are complex and span all stages of research and development, in both academic and industry environments. The basic four hurdles comprise a lack of translatability of pre-clinical research to human trials; an absence of robust prognostic and predictive biomarkers for therapeutic outcome; a need for a clear regulatory path addressing ASI modalities; and the limited acceptance to develop therapies intervening at the pre-symptomatic stages of disease. The core theme to address these challenges is collaboration—early, transparent, and engaged interactions between academic labs, pharmaceutical research and clinical development teams, advocacy groups, and regulatory agencies to drive a fundamental shift in how we think and treat T1D.

## Introduction

Type 1 diabetes (T1D) is an autoimmune disease characterized by T-cell-dependent immune destruction of insulin-producing beta-cells, leading to dysregulated glucose homeostasis. T1D is triggered by complex genetic and environmental factors, progressing from asymptomatic to autoantibody-positive to overt dysglycemia. Since the 1920s, people diagnosed with T1D have had few options beyond exogenous insulin therapy. While the ever-evolving insulin formulations and pump systems can provide automated dosing and monitoring, these only treat a symptom of the disease, not the underlying pathophysiology. This is underscored by the fact that individuals with T1D still have a reduced life expectancy compared to the general population (1) and are not relieved of their disease management.

**Figure 1:**
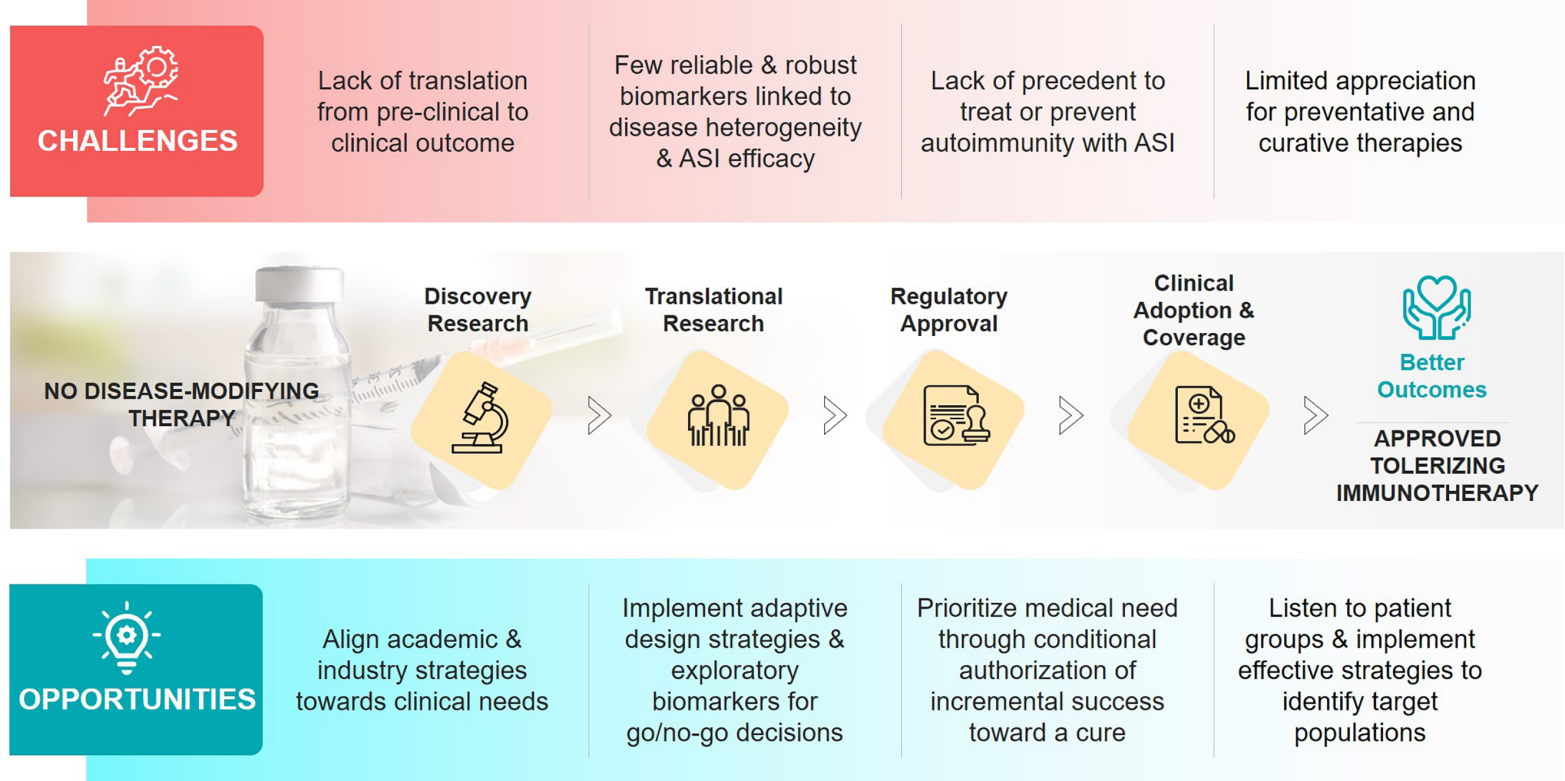
Challenges and opportunities in the development of antigen-specific immunotherapies (ASIs) for T1D.

Non-antigen-specific immunotherapies, including cytokine blockade, inhibition of T-cell co-stimulation, selective immune cell depletion, and induction of polyclonal regulatory T-cells, have targeted features of T1D-related autoimmunity, not the loss of self-tolerance (2). Although these immunotherapies have improved the management of some autoimmune diseases, none have been approved for T1D. We propose that, to achieve a significant clinical impact in T1D, we need antigen-specific immunotherapies (ASI) that work in the pre-dysglycemic stage into the early insulin-requiring period of disease, preventing and reverting overt disease manifestations. In addition, ASI might be beneficial to individuals with long-duration T1D who have residual beta-cell function (3–5). These therapies, possibly in combination with regenerative approaches, could restore glucose homeostasis by reducing the autoinflammatory pressure. ASI include various immunoregulatory formulations of proteins or peptides +/- adjuvants; plasmid-based therapies encoding multiple antigens +/- immune modulators; and antigen-presenting cell-based and engineered antigen-specific T-cell-based therapies [reviewed in (6, 7)] and some peptide, protein, and DNA-based ASI approaches have been tested in T1D [reviewed in (8)]. ASIs require a high safety profile concomitant with exquisite target/organ specificity that does not compromise host responses to pathogens or elevate cancer risks. ASIs have been challenging to develop, there has been limited clinical success, and none are approved for any autoimmune disease. The underlying challenges required to drive success are complex and involve aspects of the translatability of pre-clinical research; the lack of robust prognostic and predictive biomarkers reflecting the heterogeneity of T1D; and the hesitancy to develop treatments to intervene with the disease prior to clinical diabetes onset (**Figure 1**). Nevertheless, ASI hold such potential to transform the treatment and prevention of T1D that we believe we are at a pivotal moment to solve these challenges and progress towards solutions for prevention and cure.

## Key Challenges

### Translatability of Pre-Clinical Research

The identification of autoantigens driving T1D-specific B- and T-cell responses is rooted in human data, and further defined and evaluated in pre-clinical models [reviewed by (8, 9)]. However, pre-clinical studies evaluating the prognostic, diagnostic, and therapeutic relevance of T1D-associated autoantigens have often been conducted in the absence of a strong drug development context. As such, there is no consensus on the strategies required to demonstrate the efficacy of ASI. The learnings from animal models are limited; their translatability has been a challenge due to underlying MHC differences and the diversity of autoantigen responses in different models. Additionally, no animal model provides translatable insight into the many complications that occur over time in people with life-long T1D or into the long-term benefits of ASI.

Further, the development path of ASI may differ depending on the age range of the target population due to different requirements in antigens, therapeutic combinations, and administration frequencies. And finally, understanding the similarities and differences in the immune response and treatment of pediatric- versus adult-onset T1D is critical to the long-term success of ASI but challenging to model.

### Lack of Robust Prognostic and Predictive Biomarkers Reflecting the Heterogeneity of T1D

Currently, islet autoantibodies are the only prognostic biomarker for T1D. Standard tests measure glutamic acid decarboxylase 65 (GAD), insulin (IAA), protein tyrosine phosphatase islet antigen-2, and zinc transporter 8-specific antibodies. Their appearance predicts that clinical disease is almost certain within one’s lifetime; however, they do not predict the timing of an individual’s progression to disease (10). Autoantibodies are a hallmark of T1D, regardless of the symptomatic manifestations, yet they are not causal or informative for immediate therapeutic outcome. However, distinct T1D endotypes, driven by HLA-DRB1*03 or HLA-DRB1*04, have recently been associated with the selective appearance of GAD or IAA, respectively as the first detected autoantibody (11). These endotypes may impact the selection of tolerizing antigens and narrow patient selection for trials of peptide-based ASI, as suggested in recent trial reports (12, 13).

Autoantibody formation is preceded by pancreatic antigen-specific T-cell responses (14). However, autoreactive T-cell frequencies are highly variable in people with T1D and are also present in healthy subjects (15, 16). Though technology is rapidly advancing, especially for molecular assessment of T-cell repertoires, autoreactive T-cell-specific biomarkers suitable to predict efficacy of ASIs in T1D remain a significant roadblock. The lack of a biomarker toolkit to evaluate disease initiation and regression further challenges the design of efficient, rapid experimental studies or early clinical trials that could inform larger, phase 2 and 3 clinical trials. Although there are many reasons for this, the lack of access to the pancreas and limited data connecting peripheral immune biomarkers to beta-cell-related autoimmunity are key drivers. This challenge further hinders the identification of appropriate surrogate endpoints for clinical development of ASI in T1D (17). Ideally, predictive biomarkers for ASI should have high prognostic value for a positive clinical response outcome. For example, phenotypic changes in disease-related peripheral T cells or other surrogate markers should anticipate meaningful clinical outcomes such as reduced risk of clinical disease onset, delayed disease progression, partial or full remission, and/or reduced long-term complications (18). Without surrogate biomarkers, prevention trials may be prohibitively long (>10 years) and expensive and require that industry take on a higher risk of failure than is required with more conventional therapies. Moreover, the lack of method harmonization and of sufficient longitudinal samples to evaluate disease progression or treatment efficacy has hindered the development of a robust biomarker strategy to advance ASI for T1D.

### Lack of Precedent for Treating Autoimmune Diseases With ASIs

T1D is a disease predominantly affecting children, with a notable pre-clinical asymptomatic period. The availability of life-saving insulin and more recent, novel closed loop delivery systems to treat dysglycemia has diminished the sense of urgency to find a cure or prevention for T1D. To date, no ASI approach has been approved for any autoimmune indication, and T1D is a difficult first indication in which to drive regulatory approval. ASI drug development and early phase trials are in progress in other diseases, including coeliac disease, pemphigus vulgaris, rheumatoid arthritis, and multiple sclerosis (19–21). Although it is tempting to speculate that achievements in these indications will facilitate the adoption of ASI for T1D, ASI approaches may not necessarily extrapolate from one disease to another, because antigens, disease-associated HLA, and disease mechanisms differ. Moreover, the lack of any approved disease-modifying therapy for T1D may add additional delays as there are no examples to follow.

### Reluctance to Treat Prior to Overt Diabetes Onset

There are independent, notable challenges for ASIs when discussing and defining the target product profile for T1D —a key document that defines the characteristics of an innovative drug that addresses an unmet need. It seems there is agreement that intervention early in the disease carries the highest chance of success and several trials have been undertaken in people at risk of T1D, prior to overt disease (7). However, it is not broadly accepted that T1D starts earlier than the clinical diagnosis or that preventing further progression is more efficient than trying to reverse overt disease. This is compounded by the poor consensus among drug development teams regarding the desired impact of ASI on disease outcomes. For some, the goal is to permanently cure disease using tolerizing approaches in early disease stages e.g., prior to autoantibody development. For others, the goal is to establish ‘functional tolerance’ in pre-diagnosis (stage 2) or early onset to prevent further progression. Still others aspire to consolidate or combine ASI-induced immune tolerance with other immune-modulatory or beta-cell-preserving therapeutic strategies to quell fulminant disease. This lack of consensus is a challenge for drug developers, as this may lead to misaligned expectations of what a product might achieve in a clinical trial, with implications for trial design and outcome measures.

## Key Opportunities

### Improving Translation From Bench to Bedside

To further expand and de-risk findings from pre-clinical animal models, ASIs should be evaluated in physiologically relevant human platforms to provide greater mechanistic insight and to improve clinical translatability. With increasing access to humanized mouse models, compelling mechanism of action may be demonstrable for some ASIs, especially regarding HLA restriction of antigen and responding T cells (22). In such cases, investigational new drug (IND) applications may not need to include a demonstration of efficacy in a disease-relevant pre-clinical model (23). Furthermore, *ex vivo* tissue systems or organoids are becoming catalysts for the translation of ASIs, providing a bridge between pre-clinical and clinical studies. ASI-related researchers should take advantage of these biomimetic models whenever possible to test their therapeutic candidates. Importantly, these systems may allow assessment of drug responsiveness using representative tissue to understand disease heterogeneity. Although the availability of validated 3D systems that recapitulate *in vivo* tissue with accessible lymphatics and vascularization is not yet a reality, it is entirely plausible that the speed of technological progress will create such opportunities in the not-too-distant future.

Key learnings can be provided through small phase 0 and phase 1 clinical trials that include extensive immunologic analyses. These human studies are increasingly common (24), and may help further refine therapeutic tolerance restoration in individuals with autoimmune disease prior to embarking on large trials. However, it is critical that such small studies are clearly presented as hypothesis- or mechanism-generating, with insufficient power to define conclusive mechanism, and not as studies evaluating efficacy, to prevent over-interpretation of study results.

Early partnering between academia and industry and across public-private consortia should become the norm to accelerate preclinical drug development by integrating core academic and industry laboratories to enable generation of strong preclinical data packages. This partnering should help validate findings in multiple orthogonal animal models, allow for confidential vetting of leads, access to GMP-grade compounds for testing, evaluation of *in vivo* mechanisms and antigens in novel *ex vivo* systems, and create public-private project teams to guide development. Such teams would include clinical experts to de-risk therapeutic candidates effectively and early. Further, pharmaceutical companies should increasingly join forces with each other, and with smaller biotech companies, to co-develop ASI programs to share the risk and financial burden during early stages of T1D drug development. These partnerships could significantly lower costs and timelines, enabling all parties to reach go/no-go decisions that allow expansion or completion of partnerships at prespecified junctures. Similarly, central topics for development, such as positions on intellectual property rights, should be discussed and agreed upon early to establish clarity for downstream activities. For real progress to occur with ASI, collaborations during the ideation and development phases of drug development programs will be critical for clinical success.

### Identifying and Developing Translational Biomarkers

The need for close collaboration between academic- and industry-based groups extends to the identification and development of ASI-relevant biomarkers. These partnerships should be initiated early in the pre-clinical phase, when ASI candidates and possible predictive biomarkers are being evaluated in animal studies and biomimetic *ex vivo* model systems. With recent technological advances, such as high dimensional mass cytometry, single-cell RNA sequencing, and T-cell receptor profiling, there are increasing numbers of composite measures that hold promise as biomarkers of progression or therapeutic effect [reviewed in (25)]. For example, detailed post-hoc analyses of peripheral immune cell samples from teplizumab and alefacept trials revealed a common “partial exhaustion” transcriptomic signature of antigen-specific CD8^+^ T-cells (26, 27). Other recent studies have identified T-cell biomarkers associated with favorable prognosis close to T1D onset (28–30). Building on these tangible advancements, promising biomarkers and methods could be optimized for costs and feasibility to be applied in large, multi-center studies and validated for clinical trial use.

Much of the ongoing biomarker identification and development work for T1D has been done by academic consortia, like the Immune Tolerance Network and TrialNet, with access to relevant patient samples and a critical mass of expertise to establish clearer assay standards and procedures. This effort should be further enhanced through expanded, early collaboration involving pharmaceutical and biotechnology company partners. Increased early-stage public-private partnerships will help drive the validation and publication of robust and feasible biomarkers and assays that are subjected to industry-level rigor to refine their context of use, clarify their limitations, and to facilitate their inclusion in clinical trials in a harmonized and standardized fashion. Early successes will then enable testing of larger panels of biomarkers for broader use, such as in patient selection or stratification, that will be critical for future ASI development programs.

### Clinical Trial and Approval Path for ASI in T1D

ASI development programs will strongly benefit from early identification of regulatory challenges and development of mitigation strategies. Consultation with clinicians, trial design experts, and statisticians should begin during late preclinical activities, and feedback from regulatory agencies must be solicited as early as possible to understand their requirements (e.g., trial design elements like sample size, outcome measures, primary and secondary endpoints, and safety outputs). These early interactions can also be an opportunity to inform regulatory colleagues on the disruptive potential of ASI to prevent or cure T1D. Importantly, clinical outcomes of ASI that extend beyond delaying onset and preserving functional beta cell mass (e.g., reduced hypoglycemic events, reduction in insulin needs, and acute or long-term complications), as well as other quality-of-life measures for the patient and their caregivers, should be discussed and captured comprehensively during clinical studies and/or in post-marketing efforts. Communication of cohesive, consistent messages from academic and industry stakeholders to regulators on the rationale and strategy guiding ASI development from preclinical to clinical testing, can be a vital catalyst for progress in this field.

Consumers (i.e., people who have or are at risk of T1D, caregivers, and clinicians) should be educated on the potential of ASIs to induce permanent change in the disease processes. A more deliberate inclusion of consumer stakeholders would be a welcome addition to academic and commercial discovery and development teams. This would involve their inclusion in grant applications, in preclinical development teams during IND package generation, within clinical study design discussions, and during evaluation of clinical study results.

Further, implementation of innovative trial designs for ASI can rapidly accelerate progress. This need is underscored by the collective outcomes from traditional disease-modifying monotherapy trials conducted in T1D to date, and by consistent feedback from clinical trial sponsors and investigators.

### Moving to Curative and Prevention Therapies

ASI have the potential to provide solutions for multiple stages of T1D. ASI could be highly efficacious in early phases of disease, when there is limited autoimmunity, but ASI may also benefit people with long-term disease, especially those with residual beta-cell function. The ASI-mediated reduction of immune and metabolic stress on the beta cells could be sufficient and/or crucial, especially in combination with regenerative approaches, to restore quiescent beta-cell function. Applying the disease staging concepts of T1D could facilitate the identification of subjects who would benefit from immunotherapies intended to delay onset of or reverse T1D and to create a meaningful population health outcome (31). At-risk subjects have been successfully identified and enrolled in trials through clinical T1D consortia, such as TEDDY, TrialNet, ADDRESS, and INNODIA. Now, there is both a need and opportunity to identify large and diverse cohorts to participate in T1D trials. In fact, two general population screening programs have launched recently, one in a research setting (PLEDGE, https://research.sanfordhealth.org/fields-of-research/diabetes/pledge) and another at a patient community-level (T1Detect, https://www.jdrf.org/t1d-resources/t1detect/). Hopefully, they will pave the way for identifying at-risk subjects appropriate for clinical testing with disease-modifying therapies, including ASI.

ASI intervention should not be considered as a binary success-or-failure option in which accomplishments are compared to “one-and-done” therapies. The induction and maintenance of peripheral tolerance to pancreatic antigens may require regular therapeutic boosting in affected individuals. These could be very well received, provided they are safe, disease-specific, and patient friendly, e.g., they are administered sparsely and/or require non-invasive delivery options. Patient-focused organizations could increase discourse and information exchange amongst diverse stakeholders of the ASI development ecosystem by organizing workshops and interactive series that are designed to incentivize and facilitate drug development efforts. Such interactions could accelerate progress across the entire T1D immunotherapy field.

## Discussion

The abundance and speed of research witnessed during 2020 to fight COVID-19 forces us to consider whether comparable efforts could be achieved to effectively eliminate T1D. No specific remedies to fight SARS-CoV-2 infection existed before the December 2019 outbreak in Wuhan. However, the pandemic triggered an unprecedented worldwide effort to develop medicines to stop, treat, and prevent this infection. Immediate approaches aimed at repurposing existing drugs; then newly customized therapies, e.g., neutralizing monoclonal antibodies, proved more successful and, remarkably, SARS-CoV-2-specific vaccines were developed in record time (32). Interestingly, to date, there are >15 different COVID-19 vaccines authorized in different parts of the world including a few innovative options using completely novel technology platforms that could revolutionize vaccine development. The take-home message is that it is possible to develop innovative and efficacious solutions in a very short time.

The therapeutic options for T1D have seen minimal advancement in the past century, and the advances in the field have been restricted to incremental modifications of insulin formulations and delivery methods. Complementary to these treatments, the scientific community has explored a variety of “repurposing” options, mostly based on the use of non-specific immune modulators that have shown limited efficacy in clinical studies to-date (33). As in the case of COVID-19, it is time to understand the huge medical need associated with T1D and to make a concerted effort to develop curative solutions directed at the root cause of the disease: a breach in immune tolerance to pancreatic antigens. ASIs hold such potential and, like the new vaccines developed against SARS-CoV-2 infection, they promise a long-awaited transformative solution for the treatment and prevention of T1D.

## Data Availability Statement

The original contributions presented in the study are included in the article/supplementary material. Further inquiries can be directed to the corresponding author.

## Author Contributions

RT, JC, JW, and SA all contributed equally to the ideation, writing, and reviewing of the manuscript. All authors contributed to the article and approved the submitted version.

## Funding

Supported by a grant from The Leona M. and Harry B. Helmsley Charitable Trust and Juvenile Diabetes Research Foundation Australia to RT.

## Conflict of Interest

JC is an employee of Novartis. JW is an employee of Novo Nordisk. RT has filed provisional patents surrounding technology and is commercializing immunotherapy for targeting DCs for antigen-specific tolerance.

The remaining author declares that the research was conducted in the absence of any commercial or financial relationships that could be construed as a potential conflict of interest.

## Publisher’s Note

All claims expressed in this article are solely those of the authors and do not necessarily represent those of their affiliated organizations, or those of the publisher, the editors and the reviewers. Any product that may be evaluated in this article, or claim that may be made by its manufacturer, is not guaranteed or endorsed by the publisher.

## References

[1] Huo L Harding JL Peeters A Shaw JE Magliano DJ. Life Expectancy of Type 1 Diabetic Patients During 1997-2010: a National Australian Registry-Based Cohort Study. Diabetologia (2016) 59:1177–85. 10.1007/s00125-015-3857-4 26796634

[2] Roep BO Wheeler DCS Peakman M. Antigen-Based Immune Modulation Therapy for Type 1 Diabetes: the Era of Precision Medicine. Lancet Diabetes Endocrinol (2019) 7:65–74. 10.1016/S2213-8587(18)30109-8 30528100

[3] Roep BO Thomaidou S van Tienhoven R Zaldumbide A. Type 1 Diabetes Mellitus as a Disease of the Beta-Cell (do Not Blame the Immune System)? Nat Rev Endocrinol (2021) 17:150–61. 10.1038/s41574-020-00443-4 PMC7722981 33293704

[4] Keenan HA Sun JK Levine J Doria A Aiello LP Eisenbarth G. Residual Insulin Production and Pancreatic Ss-Cell Turnover After 50 Years of Diabetes: Joslin Medalist Study. Diabetes (2010) 59:2846–53. 10.2337/db10-0676 PMC2963543 20699420

[5] Oram RA Sims EK Evans-Molina C. Beta Cells in Type 1 Diabetes: Mass and Function; Sleeping or Dead? Diabetologia (2019) 62:567–77. 10.1007/s00125-019-4822-4 PMC6688846 30767048

[6] Carballido JM Santamaria P. Taming Autoimmunity: Translating Antigen-Specific Approaches to Induce Immune Tolerance. J Exp Med (2019) 216:247–50. 10.1084/jem.20182287 PMC6363422 30651299

[7] Serra P Santamaria P. Antigen-Specific Therapeutic Approaches for Autoimmunity. Nat Biotechnol (2019) 37:238–51. 10.1038/s41587-019-0015-4 30804535

[8] Loaiza Naranjo JD Bergot AS Buckle I Hamilton-Williams EE. A Question of Tolerance-Antigen-Specific Immunotherapy for Type 1 Diabetes. Curr Diabetes Rep (2020) 20:70. 10.1007/s11892-020-01363-3 33169191

[9] Roep BO Peakman M. Antigen Targets of Type 1 Diabetes Autoimmunity. Cold Spring Harb Perspect Med (2012) 2:a007781. 10.1101/cshperspect.a007781 22474615 PMC3312399

[10] Ziegler AG Rewers M Simell O Simell T Lempainen J Steck A. Seroconversion to Multiple Islet Autoantibodies and Risk of Progression to Diabetes in Children. JAMA (2013) 309:2473–9. 10.1001/jama.2013.6285 PMC4878912 23780460

[11] Battaglia M Ahmed S Anderson MS Atkinson MA Becker D Bingley PJ. Introducing the Endotype Concept to Address the Challenge of Disease Heterogeneity in Type 1 Diabetes. Diabetes Care (2020) 43:5–12. 10.2337/dc19-0880 31753960 PMC6925574

[12] Alhadj Ali M Liu YF Arif S Tatovic D Shariff H Gibson VB. Metabolic and Immune Effects of Immunotherapy With Proinsulin Peptide in Human New-Onset Type 1 Diabetes. Sci Transl Med (2017) 9:eaaf7779. 10.1126/scitranslmed.aaf7779 28794283

[13] Ludvigsson J Sumnik Z Pelikanova T Nattero Chavez L Lundberg E Rica I. Intralymphatic Glutamic Acid Decarboxylase With Vitamin D Supplementation in Recent-Onset Type 1 Diabetes: a Double-Blind, Randomized, Placebo-Controlled Phase IIb Trial. Diabetes Care (2021) 44:1–9. 10.2337/dc21-0318 34021020 PMC8323180

[14] Heninger AK Eugster A Kuehn D Buettner F Kuhn M Lindner A. A Divergent Population of Autoantigen-Responsive CD4+ T Cells in Infants Prior to Beta Cell Autoimmunity. Sci Transl Med (2017) 9:eaaf8848. 10.1126/scitranslmed.aaf8848 28228602

[15] Skowera A Ladell K McLaren JE Dolton G Matthews KK Gostick E. Beta-Cell-Specific CD8 T Cell Phenotype in Type 1 Diabetes Reflects Chronic Autoantigen Exposure. Diabetes (2015) 64:916–25. 10.2337/db14-0332 PMC4557541 25249579

[16] Yeo L Woodwyk A Sood S Lorenc A Eichmann M Pujol-Autonell I. Autoreactive T Effector Memory Differentiation Mirrors Beta Cell Function in Type 1 Diabetes. J Clin Invest (2018) 128:3460–74. 10.1172/JCI120555 PMC6063477 29851415

[17] Musthaffa Y Nel HJ Ramnoruth N Patel S Hamilton-Williams EE Harris M. Optimization of a Method to Detect Autoantigen-Specific T-Cell Responses in Type 1 Diabetes. Front Immunol (2020) 11:587469. 10.3389/fimmu.2020.587469 33424839 PMC7793893

[18] Ahmed S Cerosaletti K James E Long SA Mannering S Speake C. Standardizing T-Cell Biomarkers in Type 1 Diabetes: Challenges and Recent Advances. Diabetes (2019) 68:1366–79. 10.2337/db19-0119 PMC6609980 31221801

[19] Chataway J Martin K Barrell K Sharrack B Stolt P Wraith DC. Effects of ATX-MS-1467 Immunotherapy Over 16 Weeks in Relapsing Multiple Sclerosis. Neurology (2018) 90:e955–62. 10.1212/WNL.0000000000005118 29467307

[20] Nel HJ Malmström V Wraith DC Thomas R. Autoantigens in Rheumatoid Arthritis and the Potential for Antigen-Specific Tolerising Immunotherapy. Lancet Rheumatol (2020) 2:e712–23. 10.1016/S2665-9913(20)30344-1 38279365

[21] Kelly CP Murray JA Leffler DA Getts DR Bledsoe AC Smithson G. TAK-101 Nanoparticles Induce Gluten-Specific Tolerance in Celiac Disease: a Randomized, Double-Blind, Placebo-Controlled Study. Gastroenterology (2021) 161:66–80. 10.1053/j.gastro.2021.03.014 33722583 PMC9053078

[22] Pathiraja V Kuehlich JP Campbell PD Krishnamurthy B Loudovaris T Coates PT. Proinsulin-Specific, HLA-DQ8, and HLA-DQ8-Transdimer-Restricted CD4+ T Cells Infiltrate Islets in Type 1 Diabetes. Diabetes (2015) 64:172–82. 10.2337/db14-0858 25157096

[23] Streeter HB Rigden R Martin KF Scolding NJ Wraith DC. Preclinical Development and First-in-Human Study of ATX-MS-1467 for Immunotherapy of MS. Neurol Neuroimmunol Neuroinflamm (2015) 2:e93. 10.1212/NXI.0000000000000093 25798453 PMC4360798

[24] Wagar LE Salahudeen A Constantz CM Wendel BS Lyons MM Mallajosyula V. Modeling Human Adaptive Immune Responses With Tonsil Organoids. Nat Med (2021) 27:125–35. 10.1038/s41591-020-01145-0 PMC7891554 33432170

[25] Linsley PS Greenbaum CJ Nepom GT. Uncovering Pathways to Personalized Therapies in Type 1 Diabetes. Diabetes (2021) 70:831–41. 10.2337/db20-1185 PMC7980192 33741606

[26] Diggins KE Serti E Muir V Rosasco M Lu T Balmas E. Exhausted-Like CD8+ T Cell Phenotypes Linked to C-Peptide Preservation in Alefacept-Treated T1D Subjects. JCI Insight (2021) 6:e142680. 10.1172/jci.insight.142680 PMC7934874 33351781

[27] Long SA Thorpe J DeBerg HA Gersuk V Eddy J Harris KM. Partial Exhaustion of CD8 T Cells and Clinical Response to Teplizumab in New-Onset Type 1 Diabetes. Sci Immunol (2016) 1:eaai7793. 10.1126/sciimmunol.aai7793 28664195 PMC5486405

[28] Wiedeman AE Muir VS Rosasco MG DeBerg HA Presnell S Haas B. Autoreactive CD8+ T Cell Exhaustion Distinguishes Subjects With Slow Type 1 Diabetes Progression. J Clin Invest (2020) 130:480–90. 10.1172/JCI126595 PMC6934185 31815738

[29] Narsale A Lam B Moya R Lu T Mandelli A Gotuzzo I. CD4+CD25+CD127hi Cell Frequency Predicts Disease Progression in Type 1 Diabetes. JCI Insight (2020) 6:e136114. 10.1172/jci.insight.136114 PMC7934872 33301420

[30] Musthaffa Y Hamilton-Williams EE Nel HJ Bergot AS Mehdi AM Harris M. Proinsulin-Specific T-Cell Responses Correlate With Estimated c-Peptide and Predict Partial Remission Duration in Type 1 Diabetes. Clin Trans Immunol (2021) e1315. 10.1002/cti2.1315 PMC8312239 34336205

[31] Insel RA Dunne JL Atkinson MA Chiang JL Dabelea D Gottlieb PA. Staging Presymptomatic Type 1 Diabetes: a Scientific Statement of JDRF, the Endocrine Society, and the American Diabetes Association. Diabetes Care (2015) 38:1964–74. 10.2337/dc15-1419 PMC5321245 26404926

[32] Li Y Tenchov R Smoot J Liu C Watkins S Zhou Q. A Comprehensive Review of the Global Efforts on COVID-19 Vaccine Development. ACS Cent Sci (2021) 7:512–33. 10.1021/acscentsci.1c00120 PMC8029445 34056083

[33] Coppieters K von Herrath M. The Development of Immunotherapy Strategies for the Treatment of Type 1 Diabetes. Front Med (Lausanne) (2018) 5:283. 10.3389/fmed.2018.00283 30356664 PMC6189286

